# LarGAN: A Label Auto-Rescaling Generation Adversarial Network for Rare Surface Defects

**DOI:** 10.3390/s25102958

**Published:** 2025-05-08

**Authors:** Guan Qin, Hanxin Zhang, Ke Xu, Liaoting Pan, Lei Huang, Xuezhong Huang, Yi Wei

**Affiliations:** 1 Collaborative Innovation Center of Steel Technology, University of Science and Technology Beijing, Beijing 100083, China; d202410681@xs.ustb.edu.cn (G.Q.); m202121256@xs.ustb.edu.cn (H.Z.); 2 China Guangxi Beigang New Materials Co., Ltd., Beihai 536000, China; plt6299@126.com (L.P.); hleybgxc@foxmail.com (L.H.); 13977938591@139.com (X.H.); 3 Guangxi Key Laboratory of New Materials for Special Steel, Beihai 536000, China; 4 Institute of Novel Functional Materials, Guangxi Institute of Industry and Research, Nanning 530233, China; weiyi868979@126.com

**Keywords:** generation adversarial network (GAN), image generation, data augmentation, surface defects, casting slabs

## Abstract

Insufficient defect data significantly limits detection accuracy in continuous casting slab production. This limitation arises from the data collection in fast-paced production environments. To address this issue, we propose LarGAN, a data augmentation approach that synthesizes similar and high-quality defect data from a single image. We utilize a progressive GAN framework to ensure a smooth and stable generation process, starting from low-resolution image synthesis and gradually increasing the network depth. We designed a Label Auto-Rescaling strategy to better adapt to defect data with annotation, enhancing both the quality and morphological diversity of the synthesized defects. To validate the generation results, we evaluate not only standard metrics, such as FID, SSIM, and LPIPS, but also performance, through the downstream detection model YOLOv8. Our experimental results demonstrate that the LarGAN model surpasses other single-image generation models in terms of image quality and diversity. Furthermore, the experiments reveal that the data generated by LarGAN effectively enhances the feature space of the original dataset, thereby improving the accuracy and generalization performance of the detection model.

## 1. Introduction

Casting slabs are one of the most crucial raw materials in the automotive, marine, and aerospace industries, among others [1]. Their quality directly impacts the final performance of industrial products. During the production process, various defects often emerge on the slabs’ surfaces due to factors such as processing technology and rolling equipment, including scratches, cracks, and others [2]. If these surface defects are not promptly detected and addressed, the resulting steel may exhibit serious quality issues. In the past, many enterprises employed manual visual inspection to detect surface defects on slabs. While this method may have been straightforward, it suffered from poor real-time performance and detection efficiency, along with a high labor intensity and an unfavorable working environment [3].

In recent years, deep learning approaches, particularly convolutional neural networks (CNNs), have gained attention in the field of casting slab surface defect detection. An increasing number of researchers have employed machine learning algorithms to address the limitations of traditional manual visual defect detection. Ke, XU et al. [4] collected continuous casting slab datasets on-site and proposed a new feature extraction method based on curvelet transform and kernel locality preserving projection (KLPP), using SVM for sample set classification, realizing the detection and classification of cracks. Zhao et al. [5] proposed a method for surface defect extraction and description for a continuous casting slab (CC slab) which provided conditions for defect detection. Wenbo and Xu [6] chose EfficientNetB0 as the backbone framework of their target detection network, which significantly reduced the memory usage of the model, shortened the model reasoning time, and simultaneously improved the model detection accuracy. The application of deep learning methods in the industrial sector has been rapidly increasing [7,8,9,10,11,12], particularly in the fields of defect detection and equipment fault diagnosis. However, limited defect data has long been a challenging problem in the industrial domain. To address this issue, several studies have proposed various solutions. The direct and effective methods utilize generative networks for data augmentation [13,14,15]. By leveraging generative networks, the dataset can be substantially expanded, enabling the more comprehensive training of downstream models. Several studies have explored the direct application of GAN frameworks to address defect detection challenges [16]. Mohammed, S. S. et al. [17] worked to verify the effect of DCGAN, CycleGAN, and STYLEGAN3 for the high-resolution image generation of semiconductor wafer dicing-induced defects. Hu, Z et al. [18] innovatively integrated the existing U-Net and PatchGAN architectures into the CGAN framework to more effectively address the problem of data sparsity and class imbalance in fabric defect data enhancement, thereby improving the performance of their defect detection models. Zhang C et al. [19] proposed a dual-architecture generative network with an additional local generator that focuses on small, localized regions rather than the global image structure, ensuring detailed texture and color characteristics in the generated patches; Lian J et al. [20] developed a defect-amplifying generative network that synthesized enlarged defect samples from identified micro-defects to augment training data for improved detection. These efforts aim to enhance model performance and applicability under limited data availability.

Recent advances in deep learning have introduced single-image generation (SIG) [21,22], which provides a new research direction for GAN training on small datasets. The objective of a single-image training model is to capture the content information distribution of a single image and generate high-quality approximate images that closely resemble the original image. InGAN [23] proposed the first conditional single-image generation model using an encoder–decoder architecture that produces natural-looking geometric transformations of images. SinGAN introduced an unconditional single-image generation model comprising a fully convolutional generator and discriminator that leverages the Laplacian pyramid structure to learn multi-scale information. Several extensions have been developed for SinGAN, including ConSinGAN [24], which enhances the training strategy of PGGAN [25] to improve the generation quality of SinGAN through the parallel training of multiple stages. MoGAN [26] improved upon SinGAN by manually marking the image regions of interest and synthesizing them with other regions of the image to create a harmonious picture. GP-GAN [27] proposed an image-patch-matching module to accelerate the generation speed of SinGAN and expand its application space beyond simple image generation. One-shot GANs [28] are end-to-end generation models with multiple discriminators, in contrast to the multiple generators of SinGAN. A one-shot GAN can also learn different image features, thereby providing an advantage. ExSinGAN [29] first combined the inversion of a GAN and perceptual loss [30] into SinGAN to improve its performance on nontextured images.

SIG has made it possible for GANs to be applied in scenarios of data shortage, allowing for data augmentation not only for large-sample datasets, but also for small-sample datasets. Although some excellent SIG models have seen good application and improvement, there are still many issues that need to be addressed. SinGAN and its variants suffer from a significant problem in which generating results often produces chaotic structures when the models are only given input images with different scales, resulting in significant inconsistencies in the semantics and structures of the images. Therefore, to use single-image generation models on rare surface defect data, it is necessary to ensure that the generated defect regions are recognizable. Research on various generative networks typically relies on large datasets to ensure that the models comprehensively learn the underlying data distribution, thereby improving the generation of new data. However, this approach incurs significant computational costs and struggles to learn effectively from extremely rare defect data.

Therefore, we propose a novel Generative Adversarial Network called LarGAN. Our innovation lies in the optimization of the redundant structure in a progressive framework, which reduces the training time of the SIG model. By organically embedding label self-scaling into a progressive framework, LarGAN achieves clearer generative objectives during the training process. We set adjustable parameter scaling and learning rate parameters, allowing for more flexible adjustments to the generated results. We hope to use only a small number of pictures to generate real and different defect pictures for data enhancement so that the existing classic detection models can achieve better accuracy.

In our experiments, LarGAN generated images similar to actual defects for each input image, ensuring quality while preserving accurate semantic information. We also validated the usefulness of the generated images for training YOLOv8 and quantified the impact of these images on the accuracy of YOLOv8.

## 2. Methodology

The objective of this study was to propose a conditional Generative Adversarial Network for rare surface defect data that was capable of generating defect images by incorporating both images and their label information. Unlike traditional GANs, which typically require extensive training data comprising defect images to learn statistical information and generate plausible pictures, our proposed approach can generate images that are similar to the original distribution with a much smaller amount of training data. This novel methodology enables more effective defect image generation and demonstrates superior performance in learning from rare or scarce datasets.

### 2.1. Progressive Framework

In surface-detection image-generation tasks, defect images are of various sizes, and frequently adjusting the input and output sizes of the network can adversely affect the quality of the generated images. Thus, in this study, a network design employing a method of accumulating multiple convolutional layers was used to replace the fixed-convolutional-layer networks. The generator dynamically accumulated during training, with the aim of enhancing the network’s ability to handle images of different sizes. The proposed generative model $G\left(\cdot \right)$ can be represented as a pyramid model comprising multiple generators combined together, as follows:

(1) $$G_{\mathrm{pyramid}}\left(\cdot \right)=\left\{G_{0}\left(\cdot \right),G_{1}\left(\cdot \right),\ldots ,G_{n}\left(\cdot \right)\right\}\;\;\;\;n<N$$

To better generate images based on each input image, we formed pseudo-scale image pyramids of different resolutions after the input image was scaled by tag auto-scaling, and used this as the ground truth image for each training stage. A pseudo-scale image pyramid formed by an input image $X_{\mathrm{real}}$ can be represented as a set of images *x_i_* of different resolutions, where $x_{I}$ is obtained via label auto-scaling.

(2) $$X_{\mathrm{real}}=\left\{x_{0},x_{1},\ldots ,x_{n}\right\}\;n<N$$

The input of each generator in the proposed model is random noise $z_{I}$ corresponding to the size of $z_{i}$. Original images of corresponding sizes were fed into the generator of the corresponding stage together with random noise, enabling the model to generate images of various sizes and resolutions. In each training stage, a new random noise was introduced, and the generator in the lower stage fine-tuned the weights during the later higher-stage training. However, if new noise is introduced, it can cause harmful interference with the generated results. To avoid this scenario, we used a fixed set of noise maps $Z_{\mathrm{rec}}$ to generate images, where $z^{*}$ denotes a set of initialized noise maps that remain constant and make it easier to use formulas in later sections.

(3) $$Z_{\mathrm{rec}}=\left\{z_{\mathrm{rec}}^{0},z_{\mathrm{rec}}^{1},\ldots ,z_{\mathrm{rec}}^{n}\right\}=\left\{z^{*},0,\ldots ,0\right\}\;\;\;\;n<N$$

**Figure 1:**
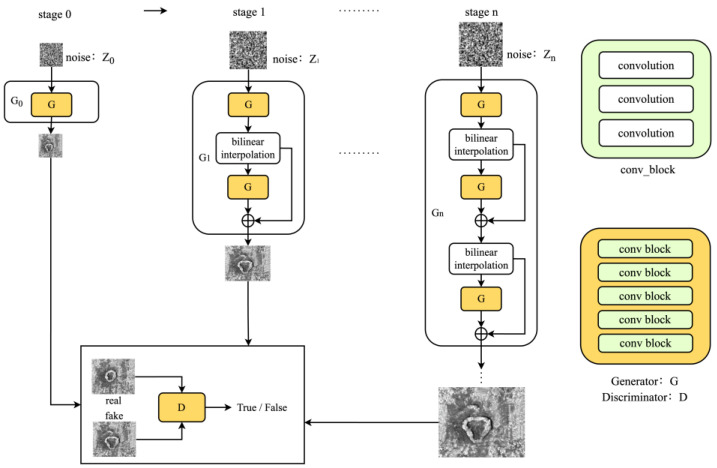
The architecture of LarGAN involves a progressive training process starting from stage 0, where the input image is initially trained at a low resolution with a small generator. As the training progresses, both the resolution of the image and the number of convolution layers in the generator gradually increases.

**Figure 2:**
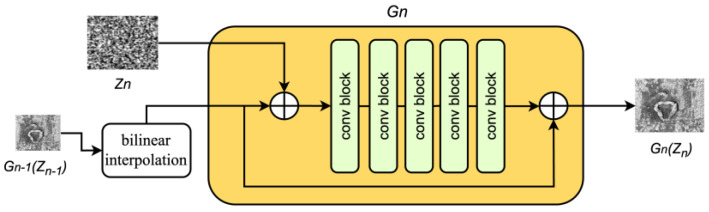
At each stage, the generator receives the generated image from the previous stage. The input image is processed by bilinear interpolation, and random noise of equal size is added. After the image passes through the five convolutional layers, a higher-resolution image can be generated.

The architecture details of LarGAN are shown in Figure 1. In this framework, $G_{n}$ represents the generator of *n*th stage, and $X_{n}$ represents the stage n. Here, n denotes the current training stage and N denotes the total number of stages to be trained. Each stage adds five convolutional layers compared with the previous stage in the network architecture of the generator, as shown in Figure 2. The structure of the discriminator D differs from that of the generator in that it does not increase the number of layers as the stages progress, but instead maintains the same parameters at each stage.

**Figure 3:**
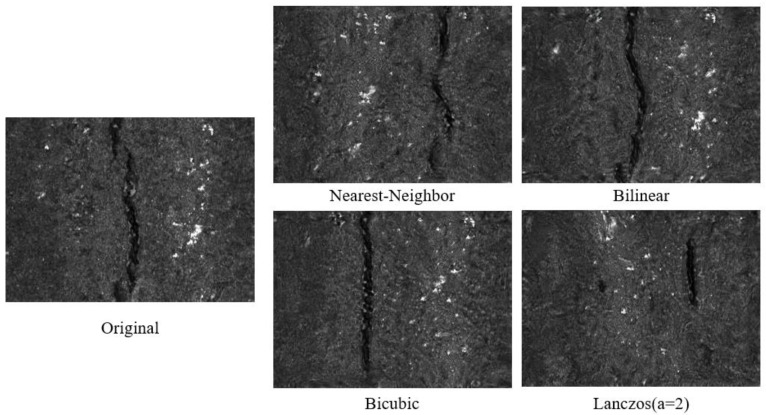
Different interpolation methods used to generate image results.

To ensure a smooth generation process, it is necessary to ensure that there are no significant changes between the adjacent stages. After comparing the results of different interpolation methods, as shown in Figure 3, we found that the nearest-neighbor interpolation method introduced noticeable aliasing artifacts and significant blurring. The bilinear algorithm improved image clarity, while the bicubic algorithm provided richer image details. The Lanczos algorithm yielded images with higher clarity and diversity. We did not choose more computationally expensive methods, such as bicubic or Lanczos interpolation, to ensure computational efficiency. Furthermore, to achieve a balance between image clarity and detail, we selected bilinear interpolation. Although bilinear interpolation may cause image distortion when significantly enlarging an image, it still performs well when applied to generation models such as LarGAN, which gradually expand images.

The loss function for the progressive framework comprised two parts: adversarial and reconstruction losses. WGAN-GP [31] was used for adversarial loss, which could effectively reduce common unstable situations in GANs, such as mode collapse, compared to other loss functions. Reconstruction loss is essentially an L2 regularization term, with the input being the real image $x^{n}$ and the generated result $G_{n}(z^{*})$ of the current stage. Given stage n, the loss function of LarGAN can be defined as follows:

(4) $$\underset{G_{n}}{\min}\underset{D_{n}}{\max}{\;L}_{\mathrm{WGAN}-\mathrm{GP}}\left(G_{n},D_{n}\right)+{\alpha ∥G_{n}\left(z^{*}\right)-x_{n}∥}^{2}$$

The value of the L2 regularization coefficient “*α*” was generally set to 10 by default, unless otherwise specified.

### 2.2. Label Auto-Rescaling

Current single-image generative networks can generate images that resemble real ones, but due to their unconditional generation nature, they typically lack effective control over the image structure and semantics, particularly in generating details and local textures. To address this issue, SinGAN and ConSinGAN propose different approaches. SinGAN generates images layer by layer, where each layer is based on the previous one and adjusted using random noise. The introduction of noise increases the diversity of the generated images, but it also makes it difficult to control the details, especially in complex image structures, leading to generated images that cannot accurately reproduce real defects or details. ConSinGAN improves upon SinGAN by adopting a multi-scale generation strategy, progressively refining each layer of the image. However, ConSinGAN focuses more on global information and does not specifically optimize local regions of the image (such as defect areas), leading to inaccuracies in defect details and unstable image quality, which may not be ideal for downstream defect detection tasks. To address these issues, we propose LarGAN, a progressive training framework based on label auto-rescaling. Unlike traditional unconditional GANs that generate images, LarGAN uses defect-area information from labels to guide the generation process. This framework progressively expands the defect areas in the labels, generating images with different resolutions and foreground-to-background ratios. Through this label-based approach, LarGAN can better control the details and semantics of the generated images, especially in defect areas, ensuring accurate and stable defect generation and providing more reliable support for subsequent defect detection tasks.

**Figure 4:**
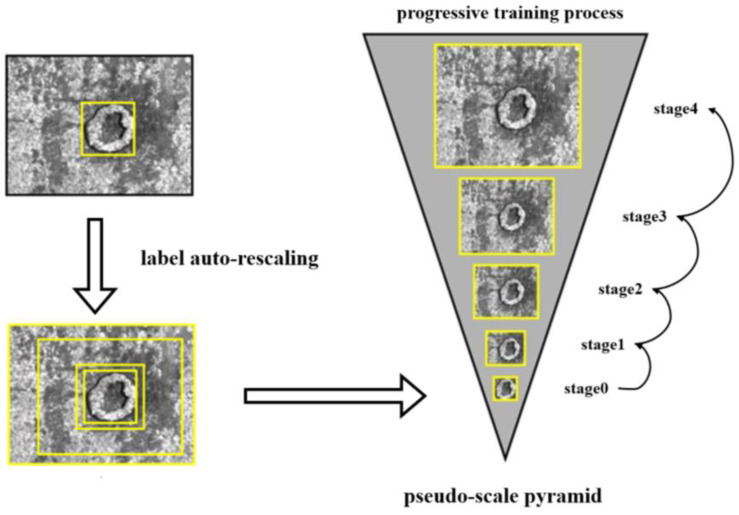
Label auto-scaling is a technique that involves providing an image as the input and obtaining a pseudo-scale pyramid as the output. Based on the image label, we specified the number of enlargements to be five, indicating the ability to restore the image from the label to its original size five times.

The label auto-rescaling method takes the defect area inside the label as the ground truth image and gradually expands it to cover the entire image, forming a pseudo-scale image pyramid with images of different resolutions and foreground-to-background ratios. As shown in Figure 4, each image in the pseudo-scale pyramid was used as the target image for that stage and compared with the images generated by the generator, gradually improving the realism of the generated images. During the training process, as the complexity of the network increased, the generator gradually learned how to produce more detailed and high-quality images.

**Figure 5:**
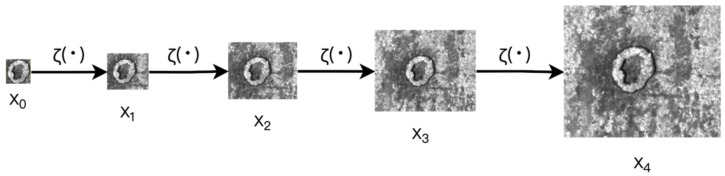
Scaling process of images with label auto-rescaling.

LarGAN is primarily designed for defect detection datasets, specifically for generating images of defective data containing labels in object detection tasks. The operation of label auto-rescaling can be defined as ζ(·), and its specific implementation details are as follows. The content inside the label box is used as the initial image $x_{n}$, which provides real images for the generator in the first stage. Together with the results of the generator $G_{0}\left(z_{0}\right)$, it is used as an input for the discriminator D to perform iterative training. Assuming that the number of progressive training stages *n* is known (which is set to the default value of *n* = 5 in hyperparameters), the scaling factor η required to restore the original image $x_{n}$ from the initial real image $x_{0}$ can be calculated. Its calculation method is detailed in Section 2.3, Rescaling Rate and Learning Rate. Finally, according to the scaling factor η, the size of the real image $x_{i}$ at each stage is calculated and saved in the pseudo-scale pyramid $x_{p\mathrm{yramid}}$. Figure 5 shows the scaling process for an image. This is the process of automatic label scaling, which takes a labeled image as input and outputs a collection of real images for training at different stages. This can be regarded as an auxiliary task for training LarGAN.

(5) $$x_{n}=\zeta \left(x_{n-1}\right)\;\;\;\;n\geq 1$$

Scaling defect regions in images to different scales and resolutions allows multi-stage generators to capture varying degrees of information. The advantage of this approach is that it avoids having the generator attempt to produce high-resolution images in the early stages of training, which can lead to training instability. Instead, by gradually increasing the size and resolution of the images, the generator can learn how to produce more stable high-quality images. Additionally, by controlling the ratio of the label area to the entire image area, the generator can primarily learn the foreground information of the image in the early stages and then improve and supplement the background information in the later stages, resulting in the desired defect images.

### 2.3. Rescaling Rate and Learning Rate

Enhancing the diversity of the generated results was crucial for introducing more defect features into the dataset. From the perspective of data augmentation, the optimal outcome was that the generated and original images were both independently and identically distributed. To ensure that the generated results from LarGAN maintain diversity and do not consistently generate the original image, we changed the way the network weights were trained. The weights trained in the previous stages were neither directly frozen nor completely re-trained. If we were to freeze the weights directly, the generated images would be similar to the original image without significant structural or morphological changes. However, if we do not freeze the weights at all, we risk losing important defect information learned in earlier stages, requiring the model to relearn the semantic information of the image during each generation, which defeats the purpose of label scaling. Therefore, we aim to control the scaling label rescaling rate and learning rate of each level of the generator to achieve the goal of generating diverse results.

To this end, we let the input images of each stage center on the defect and gradually expand outward (shown in Figure 4) up to the original image size, forming images of different sizes as a reference standard for the generated images of each training stage. The rescaling rate η was calculated based on the size of the label $\left(L_{\mathrm{label}},W_{\mathrm{label}}\right)$, the size of the image ${(L}_{\mathrm{img}},W_{\mathrm{img}})$, and the number of stages n. The rescaling rate η is a tuple ${(r}_{\mathrm{len}},r_{\mathrm{wid}})$, which represents the rate at which each stage should restore from the label size to the original image size.

(6) $$\eta =\left(r_{\mathrm{len}}{,r}_{\mathrm{wid}}\right)=\left(\frac{\sqrt[{n}]{L_{\mathrm{img}}}}{\sqrt[{n}]{L_{\mathrm{label}}}},\frac{\sqrt[{n}]{W_{img}}}{\sqrt[{n}]{W_{\mathrm{label}}}}\right)$$

Based on the rescaling rate η, the size of the ground truth image at each stage can be computed, forming a set of tuples $\left\{(\right.l_{0},w_{0}),\left(l_{1},w_{1}\right),\ldots ,(l_{n},w_{n}\left.)\right\}$. For example, if the defect image size is 160 × 120, the defect label size is 45 × 45, and the number of training stages is five, then η can be calculated to obtain (1.29, 1.22). The set of sizes of the ground truth images to be cropped at each stage is then {(45, 45), (58, 55), (75, 67), (96, 82), (124, 99)}.

The training setup was as follows: we utilized the Adam optimizer for training, with key parameters including a generator initial learning rate of 0.0005, a discriminator initial learning rate of 0.0005, and a momentum parameter of $\beta_{1}=0.5$ and $\beta_{2}=0.999$. The training process consisted of 2000 iterations, during which both the generator and the discriminator were updated three times per iteration to ensure balanced and stable adversarial training. The model was progressively adjusted through five training stages to enhance the stability of the generated images. Furthermore, the learning rate at each stage was modulated by a proportion factor ω based on the initial learning rate of 0.0005. Specifically, the effective learning rate at stage n was computed as ${0.0005\times \gamma }^{n}w$, where γ is the stage-wise decay factor. Using a fixed learning rate across all stages proved insufficient for handling defects of varying scales. Empirical results showed that a larger w (e.g., 0.8) led to greater variability in defect location and morphology, promoting the diversity of generated defects, whereas a smaller w (e.g., 0.1) preserved the general defect location while introducing subtle variations in edge and texture details. This stage-wise adjustment mechanism enabled a better balance between adaptability and diversity in defect generation. Additionally, in the multi-scale training framework, a lower scaling factor was applied to further reduce the learning rate in the higher-scale stages, facilitating finer adjustments to image details.

In the process of parameter tuning the single-image generation model, we mainly focused on four key contents: noise, learning rate, loss function, and training phase. Given the relatively small image size in our dataset of billet surface defects, no excessive training phase was required. In this experiment, we set the training phase to 5. However, for scenarios involving images with a large resolution span, it is recommended to set more training phases to prevent the generator from directly transitioning from low to high resolution, thus optimizing the refinement of image details. To avoid introducing detrimental interference in the generation results, we employed a fixed noise mapping $Z_{\mathrm{rec}}$, eliminating the need for noise adjustments. When tuning the LarGAN model, we focused on both the loss function (particularly the consistency loss coefficient *α* in Formula (4)) and the learning rate. A larger *α* (e.g., 10) encourages the generator to produce images that closely resemble the input, constraining the generator’s flexibility and thus reducing the diversity of the generated images. Conversely, a smaller *α* (e.g., 5) relaxes the consistency constraint, allowing the generator to explore a broader range of generation patterns, which increases the diversity of the generated images but may lead to deviations from the expected characteristics of the input image.

## 3. Experiments and Results

### 3.1. Data Description

**Figure 6:**
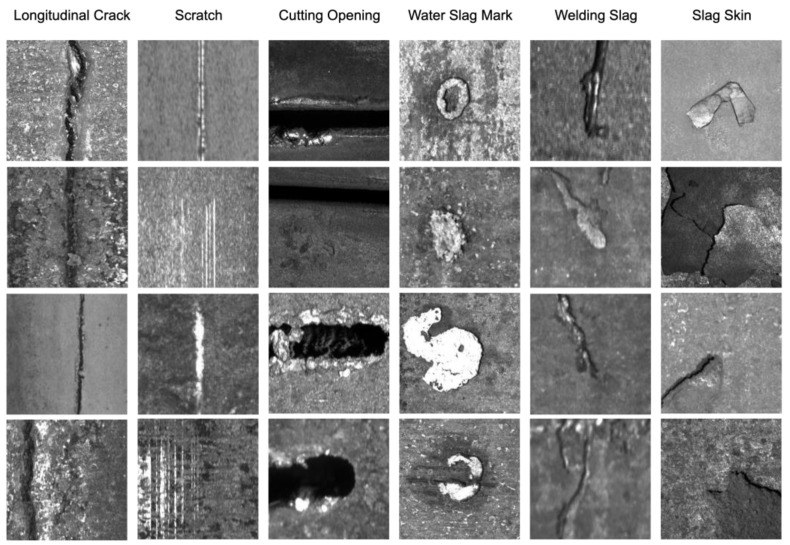
The data collection process involved gathering data from the online inspection equipment at the casting slab production site, with a specific focus on identifying and capturing 6 commonly occurring surface defects found in the cast slabs.

The dataset used in the experiments was called “Continuous Casting Slab Defects”. The defect samples were collected from the continuous casting slab surface through an online inspection system developed by Ke, XU et al. [4]. The dataset included six categories of rare defects found in casting slabs, namely longitudinal cracks (Lcs), scratches (Scs), water slag marks (WSMs), welding slag (Ws), slag skin (Ss), and cutting openings (Cos). Each category contained 400 images, resulting in a total of 2400 images of varying sizes. In Figure 6, some defect samples from each category are shown. To conduct additional experiments, we annotated the defects in the images with bounding boxes that can be used to train cutting-edge object detection networks like the YOLO series.

### 3.2. Comparisons of Quality for Generated Images

In this study, the LPIPS [32] (Learned Perceptual Image Patch Similarity) and SSIM [33] (Structural Similarity Index) metrics were utilized to evaluate the image quality generated by LarGAN. LPIPS is a metric that captures the perceptual similarity between images by leveraging deep learning to learn their perceptual features and calculating the distance between these features to quantify their perceptual similarity. SSIM is a metric used to measure the structural similarity between two images by comparing their brightness, contrast, and structural information. It assesses the similarity of images based on both structural similarity and luminance contrast similarity. We utilized these two metrics to evaluate the quality of the individual generated images.

LPIPS (Learned Perceptual Image Patch Similarity) is a metric used to measure the perceptual similarity between two images. It employs deep learning to learn the perceptual features of images and calculates the distance between the perceptual features of the two images, thereby quantifying their perceptual similarity. The calculation formula of LPIPS is as follows:

(7) $$LPIPS(I,J)=\sum_{i}∥{\phi ({I)}_{2}-\phi ({J)}_{2}∥}_{2}$$

LPIPS(I,J) represents the learned perceptual image patch similarity between images I and J. $∥{\cdot ∥}_{2}$ denotes the L2 norm (Euclidean distance). ϕ(I) and ϕ(J) are the feature representations of images I and J, respectively, obtained from a pre-trained deep neural network. A smaller LPIPS value indicates a higher perceptual similarity between the two images, meaning they are visually closer. This metric is highly useful for evaluating the performance of image generation models as it captures the perceptual similarity between images beyond just pixel-level differences. Therefore, LPIPS is widely used in tasks such as image generation and style transfer to assess the quality and realism of generated results.

SSIM is a metric used to measure the structural similarity between two images by comparing their brightness, contrast, and structure information to evaluate their similarity. The formula for calculating SSIM is as follows:

(8) $$SSIM\left(I,J\right)=\frac{\left(2\mu_{I}\mu_{J}+c_{1}\right)\left(2\sigma_{IJ}+c_{2}\right)}{\left(\mu_{I}^{2}+\mu_{J}^{2}+c_{1}\right)\left(\sigma_{I}^{2}+\sigma_{J}^{2}+c_{2}\right)}$$

$SSIM\left(I,J\right)$ represents the Structural Similarity Index between images I and J. $\mu_{I}$ and $\mu_{I}$ are the average pixel values of images I and J, respectively. $\sigma_{I}$ and $\sigma_{J}$ are the standard deviations of the pixel values in images I and J, respectively. $\sigma_{IJ}$ denotes the covariance of pixel values between images I and J. $c_{1}$ and $c_{2}$ are constants to avoid division by zero. The SSIM value ranges from −1 to 1. When the SSIM value is close to 1, it indicates a high structural similarity between the two images. Conversely, when the SSIM value is close to −1, it indicates a low structural similarity. When the SSIM value is close to 0, it means there is no structural similarity between the images.

**Figure 7:**
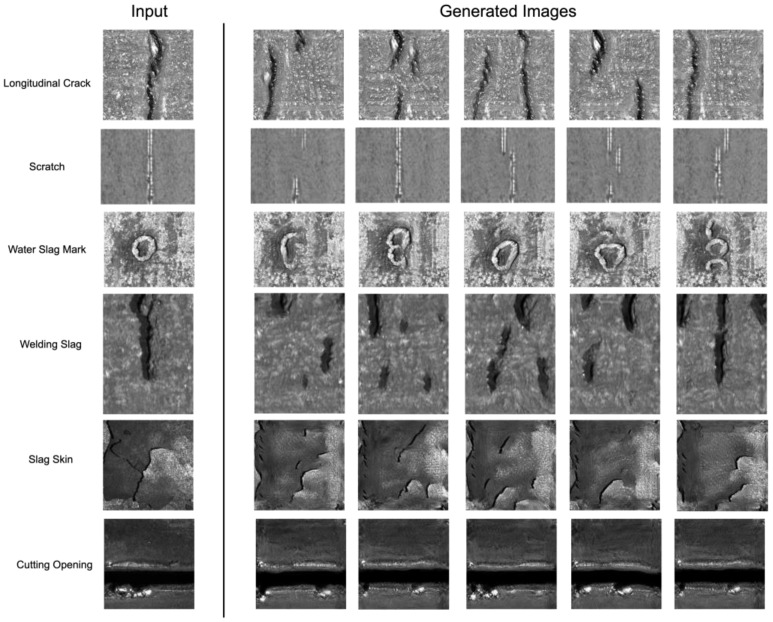
The generated results from LarGAN, when compared to the original inputs, exhibit a transformation in the appearance of defects while successfully avoiding the chaotic structures commonly observed in other GANs.

We refrained from comparing LarGAN with numerous other generative models as it would not be suitable for our specific research focus. While GANs, VAEs, and flow models often require extensive datasets for training, our primary goal is to tackle the generation of single-surface defect images to facilitate data augmentation and reduce the dependence of training detection models on data availability. Thus, fundamentally, they fall into different categories of tasks. To align with our research objectives and maintain consistency, we chose to benchmark LarGAN against SinGAN and ConSinGAN, two exemplary single-image generation models that have demonstrated remarkable performance. The performance of LarGAN was compared with two other single-image generation models, ConSinGAN and SinGAN, based on the LPIPS and SSIM metrics. Their generated results were evaluated against the original images. We showcase the images generated by LarGAN in Figure 7.

In Table 1 and Table 2, the experimental results are presented for the evaluated models (SinGAN, ConSinGAN, and LarGAN) using the SSIM and LPIPS metrics. The table provides a comparative analysis of the performance of each model based on these evaluation metrics. Regarding the LPIPS metric, both LarGAN and ConSinGAN demonstrated their respective advantages in different defect categories, while both models surpassed SinGAN. Based on the LPIPS and SSIM metrics, it can be concluded that LarGAN achieves superior image quality. Furthermore, a human perception experiment was conducted in which participants compared images generated by different models and rated their naturalness. The results showed that the generated results from LarGAN were considered the most realistic and natural, further demonstrating its perceptual advantages.

### 3.3. Comparisons of Generated Dataset Diversity

Fréchet Inception Distance (FID) is a commonly used metric for evaluating the similarity between two sets of images based on the statistics of their feature representations extracted from a pre-trained inception network. In practice, the distance between the multivariate Gaussian distributions of the real and generated images in the feature space is computed by FID. A higher degree of similarity between the two sets of images is indicated by a lower FID score. The formula for calculating FID is as follows:

(9) FID2=∥μr−μg∥2+Tr(∑r−∑g−2(∑r∑g)12)

FID represents the Fréchet Inception Distance. $\mu_{r}$ is the mean of the feature representations of the real images in the feature space. $\mu_{g}$ is the mean of the feature representations of the generated images in the feature space. $\sum_{r}$ is the covariance matrix of the feature representations of the real images. $\sum_{g}$ is the covariance matrix of the feature representations of the generated images. Tr(·) denotes the trace of a matrix. $∥\cdot {∥}^{2}$ represents the squared L2 norm. The lower the FID value, the closer the distribution of the generated images is to the distribution of the real images in the feature space. In other words, a smaller FID indicates the better quality and similarity of the generated images to the real images.

In Table 3, the performance of LarGAN was compared to two other single-image generation models, ConSinGAN and SinGAN, based on the FID metric. The experimental results showed that LarGAN achieved a significantly lower FID score of 64.3 compared to ConSinGAN and SinGAN, indicating that the images generated by LarGAN were more similar to the real images. This suggests that LarGAN is a superior model for generating high-quality and realistic images.

**Figure 8:**
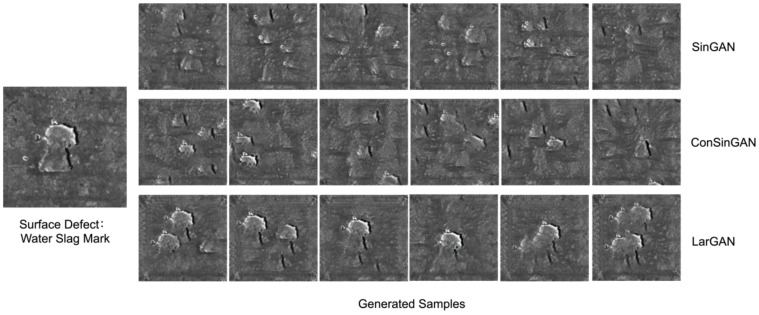
Using the proposed generative model LarGAN, the dataset is augmented to different extents, and the FID values are compared with the original dataset. The FID values exhibit that the defect image generated by Largan is more similar to the original data.

Figure 8 visually demonstrates the generated data from the three models, namely SinGAN, ConSinGAN, and LarGAN. We employed an identical defect image as the input for these three single-image generation (SIG) models and carefully compared their respective outputs. In the generated samples produced by SinGAN, a notable absence of similar defect features was observed. As for ConSinGAN’s outputs, although some images did exhibit certain defect-like features, we found these characteristics to be incomplete and remarkably unrealistic, rendering them unsuitable as labels for downstream tasks. Conversely, LarGAN’s generated data not only preserved the authenticity of the defects but also retained their comprehensive features, thus contributing a more substantial pool of plausible and utilizable new samples to the dataset. This enhancement offers valuable advantages for subsequent tasks.

**Figure 9:**
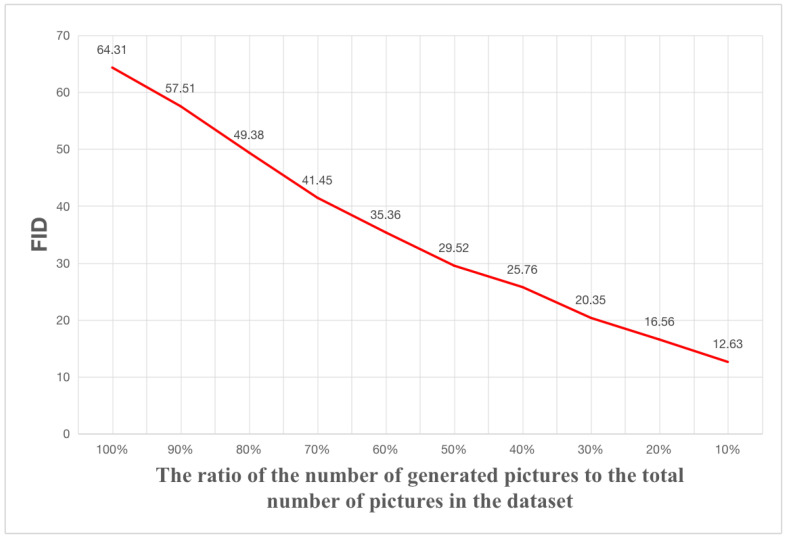
Using the proposed generative model LarGAN, the dataset is augmented to different extents, and the FID values are compared with the original dataset. The FID values exhibit a negative correlation with the proportion of generated images.

The performance of LarGAN regarding the size of the dataset was also investigated by evaluating its FID score [34] on datasets containing varying numbers of generated images, as shown in Figure 9. To assess the impact more accurately, we first tested the FID score between the 100% generated data and the original dataset, and then repeated the evaluation by reducing the proportion of generated images by 10% and adding 10% of randomly selected original images. As expected, the experimental results demonstrated a smooth positive correlation between the FID score and the proportion of generated images, indicating that increasing the dataset size could effectively enhance the generative performance of the LarGAN model.

### 3.4. Improved for Object Detection Task

It is hoped that LarGAN, the generative model we proposed, not only satisfies various standards for generative models but can also be applied to practical industrial scenarios as an auxiliary tool for defect detection. In view of the high cost of collecting rare defect samples, it is hoped that the defect data generated by LarGAN can effectively expand the sample size of datasets and maintain an independent and identical distribution with the original data, thereby improving the accuracy of detection models.

**Figure 10:**
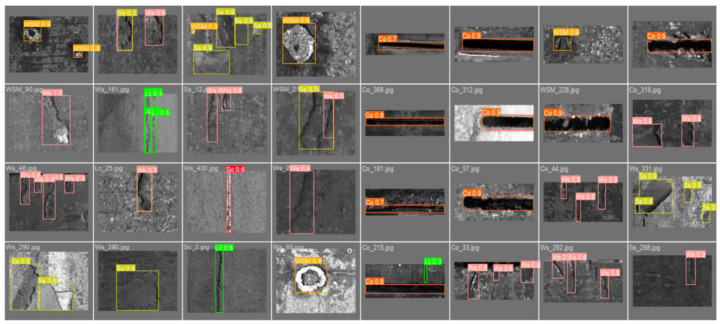
The detection results of the trained model after data augmentation are presented, revealing confidence levels exceeding 0.9 for 6 defect categories. Green, red, orange-yellow, pink, yellow, and reddish-orange boxes represent longitudinal cracks, scratches, water slag marks, welding slag, slag skin, and cutting openings, respectively.

To validate this idea, the latest detection model, yolov8, was employed and trained on 2400 images, with 1440 images for the training set, 480 images for the validation set, and 480 images for the test set. Multiple trainings were conducted with the original dataset, and the highest mAP result of 84.4 was selected as the baseline. Different proportions of generated and original images were compared with the original dataset, using the same number of images for the training set. This was conducted in order to simulate real scenarios with varying degrees of rarity of defect samples and to evaluate the performance of the generated data from LarGAN on datasets with different degrees of rarity. The optimal detection results are showcased in Figure 10, demonstrating confidence scores of up to 90% for each defect category.

To demonstrate LarGAN’s effectiveness in data augmentation across different data volumes, we designed the following experiments: First, we trained the detection model using the entire dataset to establish the best result as the upper bound. After conducting five experiments with YOLOv8, we achieved a baseline accuracy of 84.4 AP. Next, while keeping the size of test dataset constant, we reduced the total size of the training and validation datasets by 10% in each round. Therefore, we trained the YOLOv8 model with nine different data amounts, and the accuracy results are shown in Table 4. On the defect datasets of continuous casting slabs, the accuracy of YOLOv8 ranged from 61.4 to 84.4 AP. As expected, detection accuracy decreased as the data volume was reduced. Then, we augmented the training dataset with images generated by LarGAN, restoring the training datasets across different groups to the same data volume, and retrained YOLOv8. The results, as shown in Table 5, demonstrate that the images generated by LarGAN significantly improved the detection model’s accuracy. In particular, for the group with only 10% of the original data, the generated images led to a notable 6.2 AP increase in model accuracy. In summary, the images generated by LarGAN can be effectively used for data augmentation, significantly enhancing the accuracy of YOLOv8.

### 3.5. Generation Results in Other Dataset

To further evaluate the generalization capability of our proposed method, we applied it to a steel pipe surface defect dataset, which contained four defect categories (Warp, External fold, Wrinkle, Scratch), with 554 defective samples and 673 non-defective samples. The images were 728 × 544 pixels in size (this dataset is publicly available at https://github.com/clovermini/MVIT_metal_datasets, accessed on 5 May 2025).

**Figure 11:**
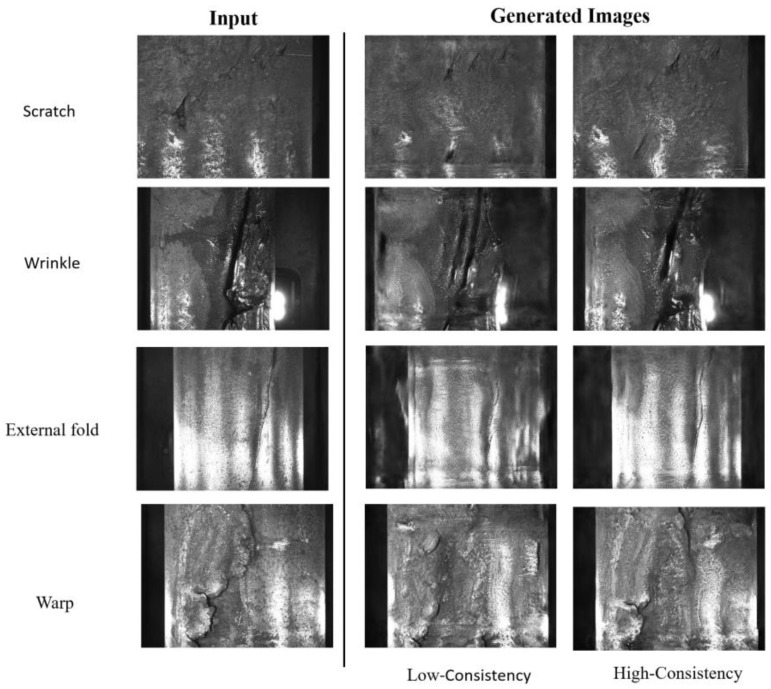
The generated results from LarGAN on steel pipe surface defect data show a transformation in the appearance of the defects compared to the original inputs, though distortions and artifacts are present at the edges of the generated steel pipes.

In this experiment, we performed defect-type generation validation on this dataset. The results show, as illustrated in Figure 11, that the generated images effectively preserved the details of the defect areas. The learning of defect information was successful, and the generated defect regions closely matched the original images, demonstrating high quality. However, we also observed that, due to variations in the diameter of the steel pipes, smaller-diameter pipes tend to have black regions at the edges, creating a straight boundary. During the generation process, even with a high consistency loss, poor edge generation was observed in some cases.

**Figure 12:**
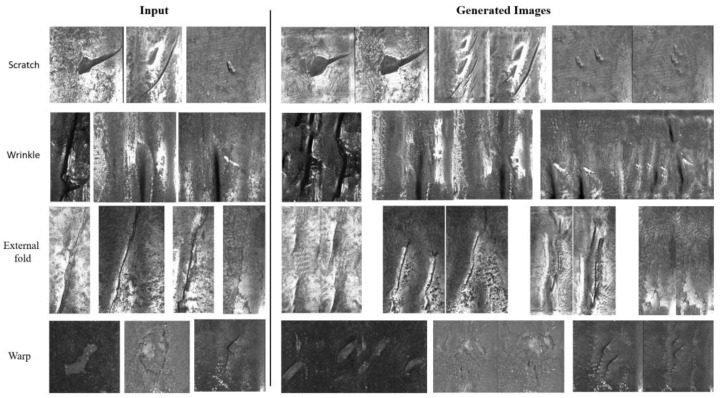
The generated results by LarGAN on the local data of steel pipe surface defects (after cropping).

To address this issue, we preprocessed the dataset by selecting local regions containing defects as the training input while reducing the input size. This not only reduced the computational load but also made the input data more similar to the surface defect data of the casting slabs, which was the focus of our original study. With this preprocessing, our generative model was still able to effectively generate defect images. Figure 12 presents the generated images from LarGAN on the steel pipe surface defect dataset. The results show that, despite the differences between this dataset and our original dataset, our method can still be successfully applied for defect data augmentation, enriching the defect sample set.

## 4. Limitations and Future Work

### 4.1. Limitation

During the experiments, we encountered a common issue—mode collapse. This problem typically manifests as overly simplistic outputs, such as pure black or yellow images. To analyze the cause of mode collapse, we examined the training data and found that it occurred more frequently when the images were generally dark and the defect features were not prominent. Based on this observation, we further investigated the underlying causes of mode collapse and concluded that, in our experiments, the primary factor was the information entropy of the input images. When the entropy of the input data is low, the generator has less information to learn from, which leads to a more limited generation mode, ultimately causing the output to degrade into its simplest form. While mode collapse can potentially be triggered by other factors, we believe that the key cause of the mode collapse observed in our experiments was the low entropy of the input data. Therefore, we suggest increasing the contrast and brightness of the images during the preprocessing stage to ensure that the input data provide sufficient feature information. This would allow the generator to fully explore the image space and avoid mode collapse due to insufficient information.

Although LarGAN performs well under standard conditions, our experiments indicate that its generalization ability is limited in more complex backgrounds. When applied to the steel pipe surface defect dataset, variations in pipe diameter and edge regions sometimes introduced background artifacts or irrelevant content in the generated images, particularly when defects were located near the pipe edges. This suggests that LarGAN does not fully account for background complexity, and further improvements are needed to enhance its robustness in diverse real-world environments. From a practical standpoint, filtering out irrelevant edge content during preprocessing can help reduce interference and improve overall performance.

### 4.2. Future Work

This work primarily addresses the issue of limited sample sizes for cast surface defects by generating single-image defect samples, thereby augmenting the defect sample pool for downstream tasks. However, while our approach has shown promising results, there are several areas for future improvement:

(1) Broader Industrial Applications: Currently, LarGAN has primarily been applied to defect data augmentation for casting slab surfaces. However, its application scope could be expanded to other industrial scenarios, such as steel, electronics, and other manufacturing sectors, providing a viable solution for defect data augmentation in a variety of fields.
(2) Multi-Defect Generation and Joint Distribution Issue: The current LarGAN method, based on a single defect sample label-scaling approach, performs well for single defect types but struggles with handling multiple defects or joint distributions of various defects. In the future, we plan to improve this by developing a multi-defect generation method to enhance LarGAN’s ability to handle complex scenarios.
(3) Enhancements in Generative Models: In addition to refining the current label scaling method, future work could explore advanced generative models, such as Diffusion Models, and methods that integrate Vision Transformers or Multimodal Large Models to further improve the quality and diversity of generated images.

## 5. Conclusions

We have developed LarGAN, a generative model that targets the scarce samples of casting slabs and which can generate defect images similar to its input images with only one defect image input. Our model is trained based on a progressive framework, and we have proposed a label auto-scaling method that adapts to this framework. By allowing the model to learn the main features of the defect first, focusing on foreground information such as defect size and shape, and then learning background information such as texture and style, LarGAN has shown advantages in both image quality and diversity compared to other single-image generation models. We have also conducted experiments on the latest object detection model, yolov8, using different training set ratios, and the results show that LarGAN can be used for data augmentation in the case of scarce samples to effectively improve the mAP of the detection model, exceeding the baseline accuracy achieved when trained on the original dataset. Additionally, LarGAN was applied to a steel pipe surface defect dataset, where it successfully learned defect characteristics from the input samples and contributed to data augmentation. The proposed LarGAN model can significantly reduce the cost of sample collection and provide an effective method for more detection models that require large amounts of data in the industry.

## Figures and Tables

**Table 1:** LPIPS for different SIG models.

Model		Our	ConSinGAN	SinGAN
Metric		LPIPS—Learned Perceptual Image Patch Similarity (Lower is Better)		
Class	Co	**0.30**	0.53	0.73
	Ws	**0.46**	0.61	0.47
	Sc	**0.37**	0.49	0.51
	Ss	0.43	**0.36**	0.69
	Lc	0.38	**0.26**	0.47
	WSM	0.40	**0.18**	0.56
	All	**0.39**	0.41	0.58

The bold values indicate the best performance among the three models for each defect type.

**Table 2:** SSIM for different SIG models.

Model		Our	ConSinGAN	SinGAN
Metric		SSIM—Structural Similarity (High is better)		
Class	Co	0.62	**0.87**	0.32
	Lc	**0.96**	0.71	0.17
	Sc	**0.92**	0.85	0.49
	Ss	**0.89**	0.67	0.47
	Ws	**0.87**	0.69	0.21
	WSM	**0.76**	0.75	0.35
	All	**0.84**	0.76	0.33

The bold values indicate the best performance among the three models for each defect type.

**Table 3:** FID for different SIG models.

Model	LarGAN	ConSinGAN	SinGAN
FID	**64.3**	73.8	104.1

The Bold value indicates the best FID score among the three models.

**Table 4:** Each time the size of the training and validation datasets is reduced by 10%, the accuracy of the model changes for the test datasets.

Experiment	Training Dataset		Validation Dataset		Testing Dataset
	Generated	Real	Generated Images	Real Images	Accuracy $({AP}_{50}$)
All Data	0	1920	0	480	83.6
90%	0	1728	0	432	83
80%	0	1536	0	384	82.1
70%	0	1344	0	336	80.3
60%	0	1152	0	288	79.9
50%	0	960	0	240	79.2
40%	0	768	0	192	76.7
30%	0	576	0	144	73.6
20%	0	384	0	96	70.5
10%	0	192	0	48	61.4

**Table 5:** The accuracy improvement of the model on the test set after adding generated data.

Experiment	Training Dataset		Validation Dataset		Testing Dataset	
	Generated	Real	Generated Images	Real Images	Accuracy $\left({AP}_{50}\right)$	Enhancement
All Data	0	1920	0	480	83.6	0
90%	192	1728	48	432	83.2	+0.2
80%	384	1536	96	384	82.4	+0.3
70%	576	1344	144	336	81.7	+1.4
60%	768	1152	192	288	81.2	+1.3
50%	960	960	240	240	80.7	+1.5
40%	1152	768	288	192	78.3	+1.6
30%	1344	576	336	144	75.4	+1.8
20%	1536	384	384	96	75.6	+5.1
10%	1728	192	432	48	67.6	+6.2

## Data Availability

The datasets generated during and/or analyzed during the current study are available from the corresponding author on reasonable request. Additionally, parts of the datasets have been made publicly available and can be accessed and downloaded at https://github.com/clovermini/MVIT_metal_datasets, accessed on 5 May 2025.

## References

[1] Yi C., Chen Q., Xu B., Huang T. (2023). Steel Strip Defect Sample Generation Method Based on Fusible Feature GAN Model under Few Samples. Sensors.

[2] Gao Y., Li X., Wang X.V., Wang L., Gao L. (2022). A review on recent advances in vision-based defect recognition towards industrial intelligence. J. Manuf. Syst..

[3] Hao Z., Li Z., Ren F., Lv S., Ni H. (2022). Strip Steel Surface Defects Classification Based on Generative Adversarial Network and Attention Mechanism. Metals.

[4] Ai Y.H., Xu K. (2013). Surface detection of continuous casting slabs based on curvelet transform and kernel locality preserving projections. J. Iron Steel Res. Int..

[5] Zhao L., Ouyang Q., Chen D., Udupa J.K., Wang H., Zeng Y. (2014). Defect detection in slab surface: A novel dual charge-coupled device imaging-based fuzzy connectedness strategy. Rev. Sci. Instrum..

[6] Xu W., Liu G., Wang M. (2023). A Deep Neural Network-Based Intelligent Detection Model for Manufacturing Defects of Automobile Parts. J. Circuits Syst. Comput..

[7] Geng Z., Shi C., Han Y. (2022). Intelligent Small Sample Defect Detection of Water Walls in Power Plants Using Novel Deep Learning Integrating Deep Convolutional GAN. IEEE Trans. Ind. Inform..

[8] Wang C., Dong S., Zhao X., Papanastasiou G., Zhang H., Yang G. (2020). SaliencyGAN: Deep Learning Semisupervised Salient Object Detection in the Fog of IoT. IEEE Trans. Ind. Inform..

[9] Zhou X., Liang W., Shimizu S., Ma J., Jin Q. (2021). Siamese Neural Network Based Few-Shot Learning for Anomaly Detection in Industrial Cyber-Physical Systems. IEEE Trans. Ind. Inform..

[10] Shao H., Li W., Cai B., Wan J., Xiao Y., Yan S. (2023). Dual-Threshold Attention-Guided Gan and Limited Infrared Thermal Images for Rotating Machinery Fault Diagnosis Under Speed Fluctuation. IEEE Trans. Ind. Inform..

[11] Cheema M.N., Nazir A., Yang P., Sheng B., Li P., Li H., Wei X., Qin J., Kim J., Feng D.D. (2021). Modified GAN-CAED to Minimize Risk of Unintentional Liver Major Vessels Cutting by Controlled Segmentation Using CTA/SPET-CT. IEEE Trans. Ind. Inform..

[12] Niu S., Li B., Wang X., Peng Y. (2021). Region-and strength-controllable GAN for defect generation and segmentation in industrial images. IEEE Trans. Ind. Inform..

[13] Li W., Zhong X., Shao H., Cai B., Yang X. (2022). Multi-mode data augmentation and fault diagnosis of rotating machinery using modified ACGAN designed with new framework. Adv. Eng. Inform..

[14] Liu S., Jiang H., Wu Z., Liu Y., Zhu K. (2022). Machine fault diagnosis with small sample based on variational information constrained generative adversarial network. Adv. Eng. Inform..

[15] Liu F., Dai Y. (2023). Product quality prediction method in small sample data environment. Adv. Eng. Inform..

[16] Abou Akar C., Abdel Massih R., Yaghi A., Khalil J., Kamradt M., Makhoul A. (2024). Generative adversarial network applications in industry 4.0: A review. Int. J. Comput. Vis..

[17] Hu Z., Schlosser T., Friedrich M., e Silva A.L.V., Beuth F., Kowerko D. Utilizing Generative Adversarial Networks for Image Data Augmentation and Classification of Semiconductor Wafer Dicing Induced Defects. Proceedings of the 2024 IEEE 29th International Conference on Emerging Technologies and Factory Automation (ETFA).

[18] Mohammed S.S., Clarke H.G. (2024). Conditional image-to-image translation generative adversarial network (cGAN) for fabric defect data augmentation. Neural Comput. Appl..

[19] Zhang C., Dai W., Isoni V., Sourin A. (2023). Automated anomaly detection for surface defects by dual generative networks with limited training data. IEEE Trans. Ind. Inform..

[20] Lian J., Jia W., Zareapoor M., Zheng Y., Luo R., Jain D.K., Kumar N. (2019). Deep-learning-based small surface defect detection via an exaggerated local variation-based generative adversarial network. IEEE Trans. Ind. Inform..

[21] Zhang Z., Liu Y., Han C., Shi H., Guo T., Zhou B. (2022). PetsGAN: Rethinking Priors for Single Image Generation. arXiv.

[22] Shaham T.R., Dekel T., Michaeli T. (2019). SinGAN: Learning a Generative Model from a Single Natural Image. arXiv.

[23] Shocher A., Bagon S., Isola P., Irani M. InGAN: Capturing and Remapping the “DNA” of a Natural Image. Proceedings of the 2019 IEEE/CVF International Conference on Computer Vision (ICCV).

[24] Hinz T., Fisher M., Wang O., Wermter S. Improved Techniques for Training Single-Image GANs. Proceedings of the 2021 IEEE Winter Conference on Applications of Computer Vision (WACV).

[25] Karras T., Aila T., Laine S., Lehtinen J. (2018). Progressive Growing of GANs for Improved Quality, Stability, and Variation. arXiv.

[26] Chen J., Xu Q., Kang Q., Zhou M. (2022). MOGAN: Morphologic-structure-aware Generative Learning from a Single Image. arXiv.

[27] Wu H., Zheng S., Zhang J., Huang K. (2019). GP-GAN: Towards Realistic High-Resolution Image Blending. arXiv.

[28] Sushko V., Gall J., Khoreva A. One-Shot GAN: Learning to Generate Samples from Single Images and Videos. Proceedings of the 2021 IEEE/CVF Conference on Computer Vision and Pattern Recognition Workshops (CVPRW).

[29] Zhang Z., Han C., Guo T. (2021). ExSinGAN: Learning an Explainable Generative Model From a Single Image. arXiv.

[30] Johnson J., Alahi A., Fei-Fei L. (2016). Perceptual Losses for Real-Time Style Transfer and Super-Resolution. arXiv.

[31] Gulrajani I., Ahmed F., Arjovsky M., Dumoulin V., Courville A. (2017). Improved Training of Wasserstein GANs. arXiv.

[32] Zhang R., Isola P., Efros A.A., Shechtman E., Wang O. (2018). The Unreasonable Effectiveness of Deep Features as a Perceptual Metric. arXiv.

[33] Nilsson J., Akenine-Möller T. (2020). Understanding SSIM. arXiv.

[34] Yu Y., Zhang W., Deng Y. (2021). Frechet Inception Distance (FID) for Evaluating GANs. China Univ. Min. Technol. Beijing Grad. Sch..

